# Diabetes Management Delivery and Pregnancy Outcomes in Women with Gestational Diabetes Mellitus during the First Wave of the 2020 COVID-19 Pandemic: A Single-Reference Center Report

**DOI:** 10.1155/2021/5515902

**Published:** 2021-07-03

**Authors:** Magdalena Wilk, Paulina Surowiec, Bartłomiej Matejko, Albert Wróbel, Joanna Zięba-Parkitny, Katarzyna Cyganek, Hubert Huras, Maciej T. Małecki

**Affiliations:** ^1^Department of Metabolic Diseases, Jagiellonian University Medical College, Krakow, Poland; ^2^University Hospital, Krakow, Poland; ^3^Students' Scientific Group, Department of Metabolic Diseases, Jagiellonian University Medical College, Krakow, Poland; ^4^Department of Obstetrics and Perinatology, Jagiellonian University Medical College, Krakow, Poland

## Abstract

**Objectives:**

The COVID-19 pandemic has forced a rapid adaptation of healthcare services to secure care for many patient groups. This includes women with gestational diabetes mellitus (GDM). We evaluated the impacts of the first COVID-19 wave on parameters such as the GDM treatment, glycemic control, and pregnancy outcomes.

**Methods:**

In this retrospective study from a reference diabetes center (Krakow, Poland), we compared patient data from two different time periods: the first wave of the COVID-19 pandemic (March 2020–June 2020) and the preceding five months (October 2019–February 2020). Data was collected from the medical records and telephone surveys.

**Results:**

We included 155 consecutive women (group N1 = 73 and group N2 = 82 from the COVID-19 pandemic period and non-COVID-19 period, respectively). During the COVID-19 pandemic, almost half of all GDM women (N1 = 36, 49.3%) used telemedicine as a method of contacting their diabetic specialists while this tool was not utilized in the earlier period. Moreover, these patients reported difficulties in performing blood glucose self-control more often (N1 = 20, 27.4%, vs N2 = 7, 8.5%; *p* ≤ 0.01) and spent less time on diabetes education than the control group on average (N1 = 39, 53.4%, vs N2 = 9, 9.8% below 2 hours of training; *p* ≤ 0.01). Most analyzed glycemic parameters and pregnancy outcomes were similar. Differences were found with respect to the incidence of prolonged labor (N1 = 12, 16.4%, vs N2 = 3, 3.7%; *p* ≤ 0.01) and preeclampsia (N1 = 0 vs N2 = 7, 8.5%; *p* = 0.01).

**Conclusion:**

In this single-center observational study, the first wave of the COVID-19 pandemic did not seem to have a negative impact on pregnancy outcomes in GDM women, despite the difficulties in diabetes management delivery.

## 1. Introduction

A novel SARS-CoV 2 virus and the resulting disease known as COVID-19 emerged in Wuhan, China, in December 2019 [1]. The first case of the coronavirus disease in Poland was recorded on March 3, 2020. This was followed by the first wave of the pandemic and triggered a national lockdown that lasted approximately three months. In Poland, SARS-CoV 2 infected nearly a million people with a total number of deaths of approximately 17000 before the end of the year [2].

The COVID-19 pandemic required the rapid transformation and adaptation of healthcare services worldwide to secure appropriate medical care for many patient groups [3]. This includes women with gestational diabetes mellitus (GDM). This condition is the most common medical complication of pregnancy and causes high-risk pregnancies [4]. It affects approximately 10% of pregnancies worldwide, and the frequency of this disease is growing systematically, especially in developed countries [5, 6]. Appropriate glycemic control during any pregnancy complicated by GDM is essential to minimize the risk of negative maternal and neonatal pregnancy outcomes [5, 7]. The key element of diabetes care is to educate on diet, self-monitoring of blood glucose (SMBG), and even insulin injections if needed [5]. Appropriate treatment modifications should be introduced without delay based on glycemic measurements. All this requires frequent outpatient visits. However, during the COVID-19 pandemic, it has been crucial to limit viral transmission through physical distancing and minimizing contact between individuals [8, 9]. Pregnant women do not appear to be more likely to contract the infection compared to the general population [10]. However, patients diagnosed with GDM are potentially at a higher risk for a severe COVID-19 infection due to predisposing factors such as hyperglycemia, frequent concomitant obesity, and hypertension. These patients also have a higher association of an increased risk of hospitalization and acute respiratory distress syndrome (ARDS) secondary to a SARS-CoV2 infection [11, 12].

Thus, there is a need for defining a model of care that would balance the necessity to prevent GDM-related pregnancy complications against limiting the risk of SARS-CoV-2 virus transmission in future mothers. At the beginning of 2020, diabetic prenatal care in GDM was rapidly redesigned worldwide to create flexible maternity care models through virtual care [12]. To secure quality of care for pregnant women with GDM, new avenues of healthcare delivery, such as telemedicine, became a commonplace in the general treatment of these patients [13, 14].

We aimed to evaluate the impact of the first wave of COVID-19 of the 2020 pandemic on the GDM treatment, glycemic control, and pregnancy outcomes.

## 2. Materials and Methods

In this retrospective study conducted at the Department of Metabolic Disease and Diabetology, a tertiary reference diabetes center at the University Hospital in Krakow, we compared two different time periods to analyze differences in treatment strategies and postpartum outcomes. The first period was between March 2020 and June 2020 and coincided with the first wave of the COVID-19 pandemic in Poland. The second period was between October 2019 and February 2020, the period five months prior to the national lockdown, to represent a control population. Patients in the first period were referred to as group 1 and represent patients who largely relied on telemedical consultations due to newly imposed SARS-CoV-2 protocols while patients in the control group and consisted of women who had concluded their pregnancy before the official lockdown in Poland on March 20, 2020, and followed more traditional GDM consultation protocols.

The universal screening for GDM has been mandatory since 1994 in Poland. The same diagnostic criteria and algorithm as well as standard of care of the Polish Diabetes Association were used in both study periods [15]. Briefly, it is recommended to perform an initial examination, usually a fasting plasma glucose, at the time of determination of pregnancy.

Women with a high risk of GDM are immediately referred for an oral glucose tolerance test (OGTT). Otherwise, the OGTT test is obligatory in the third trimester. The GDM diagnosis was made if there was at least one abnormal value (≥92, 180, and 153 mg/dl for fasting, one-hour and two-hour plasma glucose levels, respectively) during a 75g OGTT. Online demonstrations and teleconsultations became widely utilized from the beginning of the pandemic. We collected data for a retrospective analysis from medical records and telephone surveys performed with GDM patients after their delivery between August and September 2020. The study was approved by the local Bioethics Committee and conducted in accordance with the 1975 Declaration of Helsinki, as revised later.

We collected data on the specific GDM treatment, chronic concomitant diseases, the history of each patient's COVID-19 infection, the frequency of SMBG, the frequency of reported difficulties in diabetes treatment, the number of total hours devoted to in-person or online diabetic education during the pregnancy, the results of glycemic monitoring during routine visits in a diabetes clinic either in person or by telemedicine tools, and the use of auxiliary applications. The following technologies were considered telemedicine tools for this report—usually telephone consultations, e-mail contact and reports of blood glucose measurements, and rarely used video chat programs. We classified the length of GDM training into three categories—less than 2 hours, 2–5 hours, and 5 or more hours. The patients were asked to assess the quality of diabetic care using a 5-point scale, where 1 was the lowest grade and 5 was the highest.

Based on the questionnaires and available medical documentation, maternal and neonatal outcomes were assessed. The list of collected maternal outcomes included preeclampsia, obstetric hemorrhage, and postdural syndrome while neonatal end points were prolonged labor, perinatal injuries, umbilical cord wrap, hypoxia, and breeched positioning. We also collected information about the method of delivery as well as early neonatal complications after birth.

Differences between the groups were analyzed using Student's *t*-testing and nonparametric testing such as the Mann-Whitey *U* test when appropriate (Shapiro-Wilk test was used to assess normality of the distribution). To test for relationships between two categorical variables, the chi-squared or Fisher exact test was used. All statistical analyses were conducted using *STATISTICA* software ver. 13 (StatSoft Inc., USA).

## 3. Results

The characteristics of the study groups are provided in Table 1. We included 155 women with GDM diagnosis who received diabetic care in the Department of Metabolic Diseases and Diabetology at the University Hospital in Krakow between October 2019 and June 2020. There were 73 GDM patients in the COVID-19 pandemic period (group 1) and 82 in the control group (group 2), respectively. No patient in this group was diagnosed with concomitant COVID-19. The mean age of patients treated during the COVID-19 pandemic was 31.93 ± 4.15 years, and they were slightly younger compared to the controls—33.8 ± 4.5 years (*p* = 0.01). No difference was observed in the gestational week at the first visit to the diabetes clinic (group 1: 23.7 ± 7.4 weeks; group 2: 23.6 ± 8.5 weeks; *p* = 0.65). The groups did not differ significantly in terms of prepregnancy body mass index (BMI) and body weight. We did not also notice any difference in the prevalence of comorbidities such as arterial hypertension, thyroid diseases, polycystic ovarian syndrome (PCOS), and lipid abnormalities. None of the patients treated during the COVID-19 pandemic were diagnosed with the SARS-CoV2 infection.

Information concerning GDM medical care and glycemic control is shown in Table 2. During the first wave of the COVID-19 pandemic, 36 out of 72 patients (49.3%) had at least one telemedical consultation. In the control group, all patients received in-person visits with a diabetes specialist and no consultations were carried out using the telemedicine platform. During the COVID-19 period, the GDM treatment model that was the most frequently used was a diabetic diet only (36 women, 49.3%), followed by basal insulin (25 women, 34.2%), intensive insulin therapy with multiple daily injections (MDI) (12 women, 16.4%), and short-acting insulin injection(s) to selected meal(s) (1 woman, 1.4%). When analyzing the patients treated directly before the COVID-19 pandemic, no significant statistical differences were observed in the frequency of treatment as the number of patients on the respective models were as follows: diet only—29 (35.3%), basal insulin—32 (39.0%), MDI—19 (23.2%), and short-acting insulin only—2 (2.4%). No difference in the total daily dose of insulin occurred between the groups (19.35 ± 17.28 units vs 23.42 ± 20.63 units for the COVID-19 and non-COVID-19 periods, respectively, *p* = 0.57).

We also did not note a difference for the SMBG frequency. In the group of patients treated during the COVID-19 pandemic, there were no GDM patients who would measure blood glucose three times a day or less, while one patient in the group treated before the COVID-19 pandemic reported this frequency. Three to five measurements a day were used by 16 (21.9%) patients treated in the COVID-19 pandemic group as compared to 11 patients (13.4%) in the control group (*p* = 0.16). Most patients have measured glucose levels six to ten times per day: 57 patients (78.1%) in the COVID-19 grouping vs 67 patients (81.7%) in the control group (*p* = 0.57). Only three patients in the control group measured their glucose levels 11 or more times a day (*p* = 0.28).

We also compared the mean average of mean fasting glucose levels provided by the women in group 1 and the control group (87.1 ± 7.0 mg/dl vs 87.1 ± 6.6 mg/dl; *p* = 0.7) and the average of the mean postprandial glucose levels measured 60 minutes after the meal was ingested (114.9 ± 9.5 vs 117.1 ± 12.7; *p* = 0.7). No significant differences in glycemic control were observed between these groups of patients.

The most frequently reported difficulty indicated by the patients from both GDM groups was maintaining a diabetic diet (36 patients, 49.3% vs 29 patients, 35.4%; *p* = 0.08, in the COVID-19 vs control groups, respectively). However, more patients in the group studied over the COVID-19 pandemic-reported problems with SMBG as compared to the controls (20 patients, 27.4% vs 7 patients, 8.5%; *p* < 0.01). They also equally often pointed to the fear of hyperglycemia and fear of hypoglycemia as well as uncertainty regarding the technique of insulin administration and its dosing (shown in details in Table 2).

There were also differences between the groups in recording and presentation of glycemic data during a routine visit either in person or by telemedicine tools. The COVID-19 period group used a traditional paper notebook to record measurements less frequently than the controls (60 patients, 82.2%, vs 78 patients, 95.1%; *p* < 0.01). While the overall frequency of using any mobile applications for GDM patients had not increased, the patients during the COVID-19 period were more likely to use mobile applications connected to their glucometer (for example, *Contour App* and *MySugr App*) (33 patients, 45.2%, vs 23 patients, 28.0%; *p* = 0.03). As opposed to the earlier period, in the era of the COVID-19 pandemic, some GDM patients used dietary applications (7 patients, 9.6%, vs 0 patients; *p* < 0.01).

All women with GDM from both groups received training on their diet, SMBG, and insulin use. In the pandemic group of GDM patients, there were 39 women (53.4%) reporting training times as less than 2 hours, while there were only 8 such women (9.8%) in the control cohort (*p* < 0.01). Additionally, 33 patients (45.2%) indicated 2–5 hours as the duration of diabetes training, while in the control group, there were 69 such patients (84.1%) (*p* ≤ 0.01). The COVID-19-period patients reported the need for additional diabetes consultations beyond the agreed dates of visits to the Diabetes Outpatient Clinic due to the difficulties encountered in GDM treatment in 20.5% of cases (15 patients) as compared with 23.2% (19 patients) in the control group (*p* = 0.69). The assessment of diabetes care on a 1–5 scale did not differ among the patients in both groups (*p* = 0.81).

Data on maternal and neonatal outcomes are shown in Table 3. The groups did not differ in terms of the sex of the newborns. Similarly, the week of pregnancy during delivery was similar in both groups—38.5 ± 1 in the COVID-19 pandemic group vs 39 ± 2 in the controls (*p* = 0.98). The groups did not differ in terms of the prevalence of preterm births and the way of delivery, the incidence of obstetric hemorrhage or postdural syndrome. Maternal perinatal complications occurred with a similar frequency of 17.8% (13 cases) as compared to 20.7% in the control group (17 cases) (*p* = 0.65). In the COVID-19 group, there were no occurrences of preeclampsia, while in the control group, this complication occurred in 7 patients (8.5%) (*p* = 0.01).

Further, the newborns from both groups did not differ with respect to the APGAR points obtained at 5 minutes after birth and the frequency of births of children with asphyxia. Birth weight of neonates was also similar in both study groups (3217 ± 721 g vs 3252.16 ± 701 g; *p* = 0.92). The frequency of perinatal complications was also assessed. In the study group, they affected 20 children (27.4%) as compared to 31 children (37.8%) in the control group (*p* = 0.17). The most common complication in the study group was prolonged labor and it occurred statistically more often in the COVID-19 period group than in the control cohort (12 cases, 16.4%, vs 3 cases, 3.7%, *p* < 0.01). No differences were reported for perinatal complications in children and the frequency of hospitalization in the neonatal intensive care units.

## 4. Discussion

Here, we present single-center data from the retrospective analysis on diabetes care and pregnancy outcomes in GDM during the first wave of the COVID-19 pandemic in Poland. This pandemic has changed the way in which medical care is provided to many groups of patients dramatically [16]. While the numbers of affected COVID-19 patients and deaths due to this infection attracts the attention of the medical specialists, journalists, politicians, and the media, concerns are growing about the potential health consequences for patients affected by many different diseases [17]. This list also includes women with GDM. Of note, the window of opportunity for medical intervention in these female patients is narrow, usually no longer than 3 months, and the potential difficulties of access to healthcare may affect both mothers and their children.

We report some differences in medical care, including the frequency of informatics tools used both before and during the pandemic; however, it seems that no differences were observed in the complication rates of pregnancies and neonatal outcomes. First, we shall discuss how much the current pandemic influenced the provided education, treatment methods, and glucose monitoring performed by the GDM patients. It is well-proven that diabetic education plays an important role in glycemic control in all types of diabetes, including GDM [18–21].

Not surprisingly, GDM women from the COVID-19 period spent less time on diabetes training in our study. This was probably caused by the lower willingness of patients to report to the medical center in person and insufficient access to online training courses or a lack of experience in using them. Another factor could have been an individual family situation, for example, the closure of kindergarten and preschools for their children as schools in Poland were closed on March 12, 2020. Our female patients reported also a higher frequency of problems during diabetes treatment related to SMBG. Interestingly, this did not result in a lower frequency of performed glucometer measurements. Consistently, there were no differences in the number of GDM patients on the insulin treatment. Moreover, unexpectedly, a nonsignificant numerical trend was observed for the higher frequency of insulin therapy in the COVID-19 period cohort. Patients treated during the time of the first wave of the COVID-19 pandemic received a similar number of additional diabetes consultations, apart from the individual appointment schedule. So far, no other data on the impact of diabetes education of GDM women on pregnancy outcomes has been reported.

As expected, during the COVID-19 pandemic, a substantial part of consultations was delivered to the GDM patients using telemedicine. This way of performing the consultation was not used at all by the control group as all diabetic care visits and training were held in person. The situation related to the COVID-19 pandemic created a need for moving traditional face-to-face GDM education sessions to remote delivery, using mobile health tools, interactive webinars, and online resources [10]. According to the rapidly modified guidelines and recommendations for GDM care, such decisions may reduce coronavirus exposure during prenatal care and should be tailored for high-risk prenatal patients [22].

There is evidence collected over the last decade from the pre-COVID-19 era that telemedicine services improve maternal and neonatal outcomes in women with GDM [23]. Already before the current pandemic, the Canadian guidelines suggest that video or phone calls reduce face-to-face communication in initial and follow-up visits [24]. According to a recent survey performed in the US targeting the obstetrical population, more than 85% of patients desired telemedicine contact with their healthcare team between their regular face-to-face visits [13]. We can expect that after the end of the pandemic, some of these tools will be more commonly utilized by healthcare providers in order to conduct more frequent and convenient medical interactions and optimize GDM patients [25].

For some GDM patients, using such telemedicine tools may constitute a new challenge. For example, the patients from our cohort were systematically encouraged to do so by the doctor treating their GDM. Still, about 1/3 of patients did not use mobile supportive applications to improve glycemic control. It is worth noting that patients treated during the COVID-19 era tended to use mobile applications that can transmit data from a glucometer and diet applications noticeably more often. For this reason, it is probable that these same patients preferred to keep track of their data in the form of an application compared to a more traditional recording in a paper glycemic record book. In Italy, most diabetology outpatient clinics use an informatic folder tool and remote registration processes of SMBG data are being developed through specific applications [14]. Mobile phone applications are created with artificial intelligence that automatically classifies and analyzes the data (ketonuria, diet transgressions, and blood glucose values), making adjustment recommendations regarding diet or insulin treatment [26].

Our data on glycemia, mean fasting and postprandial glucose levels indicate no differences in the metabolic control of patients during the COVID-19 pandemic. One recent study from France showed that glycemic control worsened during the COVID-19 pandemic lockdown among patients with GDM in this country. The possible reasons are reduced physical activity, modified dietary habits, and greater anxiety during this period [27]. However, Italian data from the COVID-19 pandemic spring wave on a general type 1 diabetes cohort using continuous glycemic control systems showed an unexpected improvement in terms of the reduced number of hypoglycemic episodes [28]. We should also note that there were no patients affected by COVID-19 in our GDM cohort. The latest Italian report showed that there was no difference in glycemic control among patients with concomitant gestational diabetes and SARS-CoV-2 infection [29].

Finally, we should discuss the important clinical maternal and neonatal outcomes reported from our cohort. The key observation from this study is that in spite of challenges related to diabetes care, the number of unfavorable outcomes and complications did not differ between the groups. Specifically, the number of cesarean sections (C-sections) and induced deliveries did not increase, the children had a similar APGAR score, and there was no higher frequency of deliveries of premature babies and children with asphyxia. This is in line with a recent GDM study from Israel, in which a similar rate of C-sections,, including emergency procedures, was reported when comparing the COVID-19 pandemic and non-COVID-19 pandemic patients [30]. In the time of the COVID-19 pandemic, following the recommendations of gynecological specialists, surgical delivery should be reserved for usual obstetric indications [31]. As long as there is an appropriate indication and it is performed at the appropriate gestational age, scheduled C-sections should not be delayed based on just the COVID-19 pandemic [32].

In this study, there was also no overall increase in the incidence of perinatal complications; however, a higher incidence of prolonged labor was found in the COVID-19 period. Interestingly, in this group, a decrease in the frequency of preeclampsia was also observed among mothers giving birth during the COVID-19 pandemic. One may wonder whether this complication could have been simply underreported due to less intensive and efficient diagnostic procedures. The children of our GDM cohorts did not differ in their birth weight, the frequency of hospitalizations in neonatal intensive care units, and the incidence of jaundice, hypoglycemia, or hypoxia episodes.

Among the strengths of this report, one should point to providing data on not only diabetic care and glycemic data but also on maternal and neonatal pregnancy outcomes. Additionally, this group from one center was very homogeneous in terms of the offered standard of care and medical procedures. The limitation of this work is the relatively small study group and its observational nature that makes it impossible to establish causal relationships. This study was not epidemiological in nature; thus, we were not able to assess neither the regional nor the state-wide incidence of GDM and their potential impact on the study outcomes. Moreover, we cannot exclude an unlikely possibility of the significant impact of seasonal glucose variability that it may have had on our results [33]. Also, we used the date of first booking as an estimation of the date of GDM diagnosis; this, however, seems unlikely to have an impact on the study results as the visits of our GDM women are usually performed within a short time after abnormal OGTT results (usually within a few days). Additionally, this report comes from a tertiary center and the results may not be representative of the entire country. Finally, the time between the delivery and our telephone survey contact was shorter for the COVID-19 period group than for the controls and this could have influenced the reported data.

## 5. Conclusions

In summary, according to the results of this single-center observation, we report that the first wave of COVID-19 pandemic seemed not to not have caused a negative impact on glycemic control and pregnancy outcomes in GDM women, in spite of reported difficulties in diabetes management delivery.

## Figures and Tables

**Table 1:** Clinical characteristics of the study groups.

Variable	COVID-19 pandemic group N1 = 73, mean ± SD/*n* (%)	Control group N2 = 82, mean ± SD/*n* (%)	*p* value
Age at GDM diagnosis (year)]	31.9 ± 4.1	33.8 ± 4.5	0.01
Pregnancy week at the first GDM visit (weeks)	23.7 ± 7.4	23.6 ± 8.5	0.65
Body weight before pregnancy (kg)	71.3 ± 15.2	68.3 ± 16.2	0.13
Prepregnancy BMI (kg/m^2^)	26.1 ± 5.2	26.5 ± 5.6	0.51
75 g OGTT results (mmol/l)			
(i) 0 minute	5.0 ± 0.5	5.0 ± 0.6	0.86
(ii) 60 minutes	9.3 ± 1.9	9.6 ± 1.9	0.50
(iii) 120 minutes	8.5 ± 1.6	8.2 ± 1.7	0.96
Mothers' comorbidities (*n* (%))			
(i) Arterial hypertension	2 (2.7)	4 (4.9)	0.69
(ii) Thyroid disease	16 (21.9)	21 (25.6)	0.51
(iii) PCOS	1 (1.4)	4 (4.9)	0.37
(iv) Lipid disorders	1 (1.4)	2 (2.4)	0.63

**Table 2:** Diabetes medical care and glycemic control according to the study group.

Variable	Patients treated during COVID-19 pandemic N1 = 73, mean ± SD/*n* (%)	Control group N2 = 82, mean ± SD/*n* (%)	*p* value
Telemedicine use (*n* (%))	36 (49.3)	0	<0.01
GDM treatment (*n* (%))			
(i) Diabetic diet only	36 (49.3)	29 (35.3)	0.08
(ii) Basal insulin	25 (34.2)	32 (39.0)	0.54
(iii) MDI	12 (16.4)	19 (23.2)	0.30
(iv) Basal plus	1 (1.4)	2 (2.4)	0.50
Total daily insulin dose (IU)	19.3 ± 17.3	23.4 ± 20.6	0.57
Frequency of SMBG per day ((*n* (%))			
(i) 0–2	0	1 (1.2)	0.34
(ii) 3–5	16 (21.9)	11 (13.4)	0.16
(iii) 6–10	57 (78.1)	67 (81.7)	0.57
(iv) 11 and more	0	3 (3.6)	0.28
Fasting blood glucose levels (mg/dl)	87.3 ± 7.0	87.1 ± 6.6	0.70
Postprandial glycemia (60 minutes after a meal) (mg/dl)	114.9 ± 9.5	117.1 ± 12.7	0.70
Difficulties in GDM treatment (*n* (%))			
(i) Not reported	8 (11.0)	1 (1.2)	0.01
(ii) Diet	36 (49.3)	29 (35.4)	0.08
(iii) Technique of insulin administration	16 (21.9)	19 (23.2)	0.85
(iv) Insulin dosing	5 (6.8)	1 (1.2)	0.07
(v) Glycaemia self-monitoring	20 (27.4)	7 (8.5)	<0.01
(vi) Fear of hyperglycemia	23 (31.5)	22 (26.8)	0.52
(vii) Fear of hypoglycemia	3 (4.1)	2 (2.4)	0.56
Recording and presentation of glycemic data (*n* (%))			
(i) Traditional notebook	60 (82.2)	78 (95.1)	<0.01
(ii) Applications for mobile devices	36 (49.3)	27 (32.9)	0.04
Length of GDM training (*n* (%))			
(i) Less than 2 hours	39 (53.4)	8 (9.8)	≤0.01
(ii) 2–5 hours	33 (45.2)	69 (84.1)	≤0.01
(iii) 5 hours and more	1 (1.4)	5 (6.1)	0.12
Need for additional diabetic consultations (*n* (%))	15 (20.5)	19 (23.2)	0.69
Assessment of diabetic care (1–5) (*n*)	4.4 ± 0.75	4.5 ± 0.71	0.81

**Table 3:** Perinatal complications and obstetric outcomes in the study groups.

Variable	Patients treated during COVID-19 pandemic, N1 = 73, mean ± SD/*n* (%)	Control group, N2 = 82, mean ± SD/*n* (%)	*p* value
Gender of newborns (*n* (%))				
(i) Female	37 (50.7)	31 (37.8)	0.25	
(ii) Male	36 (49.3)	51 (62.2)		
Week of pregnancy during delivery (week)	38.5 ± 1	39.0 ± 2	0.98	
Preterm births (*n* (%))	7 (9.6)	14 (17.1)	0.17	
Birth weight (grams)	3217 ± 721	3252 ± 701	0.92	
Points in APGAR scale in 5′ (*n*)	9.7 ± 0.72	9.6 ± 1.33	0.51	
Occurrence of asphyxia (*n* (%))	3 (4.1)	6 (7.3)	0.39	
Maternal perinatal complications (*n* (%))	13 (17.8)	17 (20.7)	0.65	
(i) Pre-eclampsia	0 (0)	7 (8.5)	0.01	
(ii) Obstetric hemorrhage	5 (6.8)	5 (6.1)	0.85	
(iii) Post-dural syndrome	3 (4.1)	0 (0)	0.06	
(iv) Others	5 (6.8)	5 (6.1)	0.85	
Newborn perinatal complications (*n* (%))	20 (27.4)	31 (37.8)	0.17	
(i) Prolonged labor	12 (16.4)	3 (3.7)	<0.01	
(ii) Perinatal injuries	2 (2.7)	3 (3.7)	0.73	
(iii) Umbilical cord wrap	1 (1.4)	4 (4.9)	0.37	
(iv) Hypoxia	3 (4.1)	9 (11.0)	0.14	
(v) Breech position	1 (1.4)	7 (8.5)	0.07	
(vi) Others	3 (4.1)	5 (6.1)	0.45	
Way of delivery (*n* (%))				
(i) Cesarean section	37 (50.7)	45 (54.9)	0.06	
(ii) Vaginal birth	10 (13.7)	12 (14.6)	0.87	
(iii) Inducted vaginal birth	26 (35.6)	25 (30.5)	0.50	
Early complications after birth (*n* (%))				
(i) No complications	33 (45.2)	33 (40.2)	0.53	
(ii) Jaundice	27 (36.9)	38 (46.3)	0.24	
(iii) Hypoglycemia	12 (16.4)	9 (11.0)	0.32	
(iv) Breathing disorders	5 (6.8)	4 (4.9)	0.61	
Neonatal intensive care unit hospitalization (*n* (%))	11 (15.1)	11 (13.4)	0.77	

## Data Availability

All data are available upon request from the authors.
